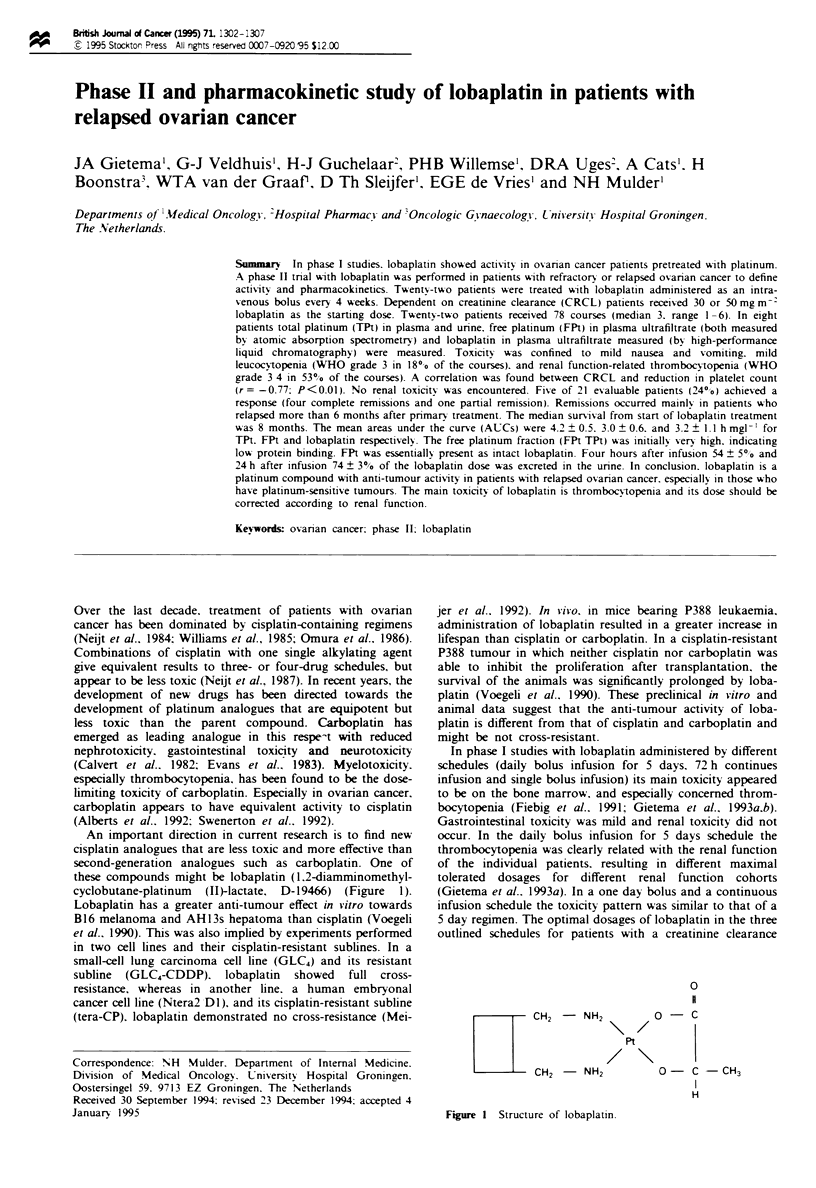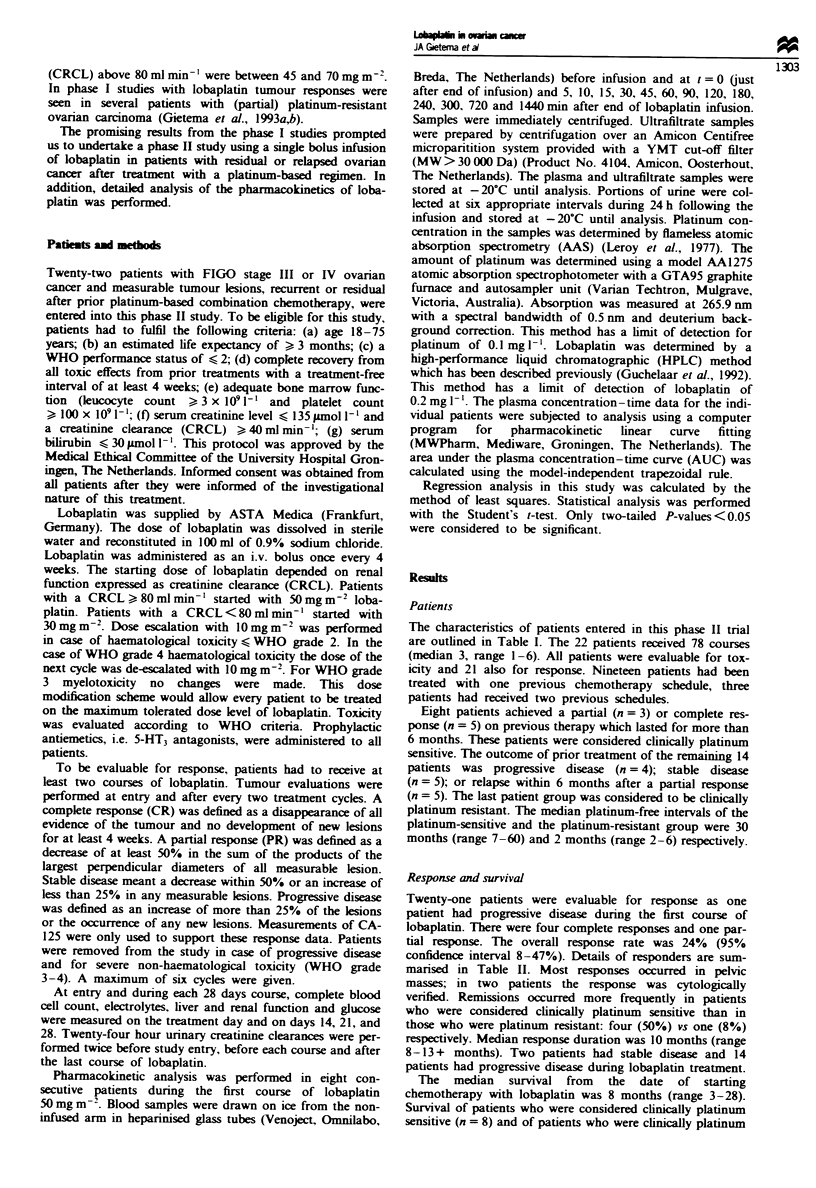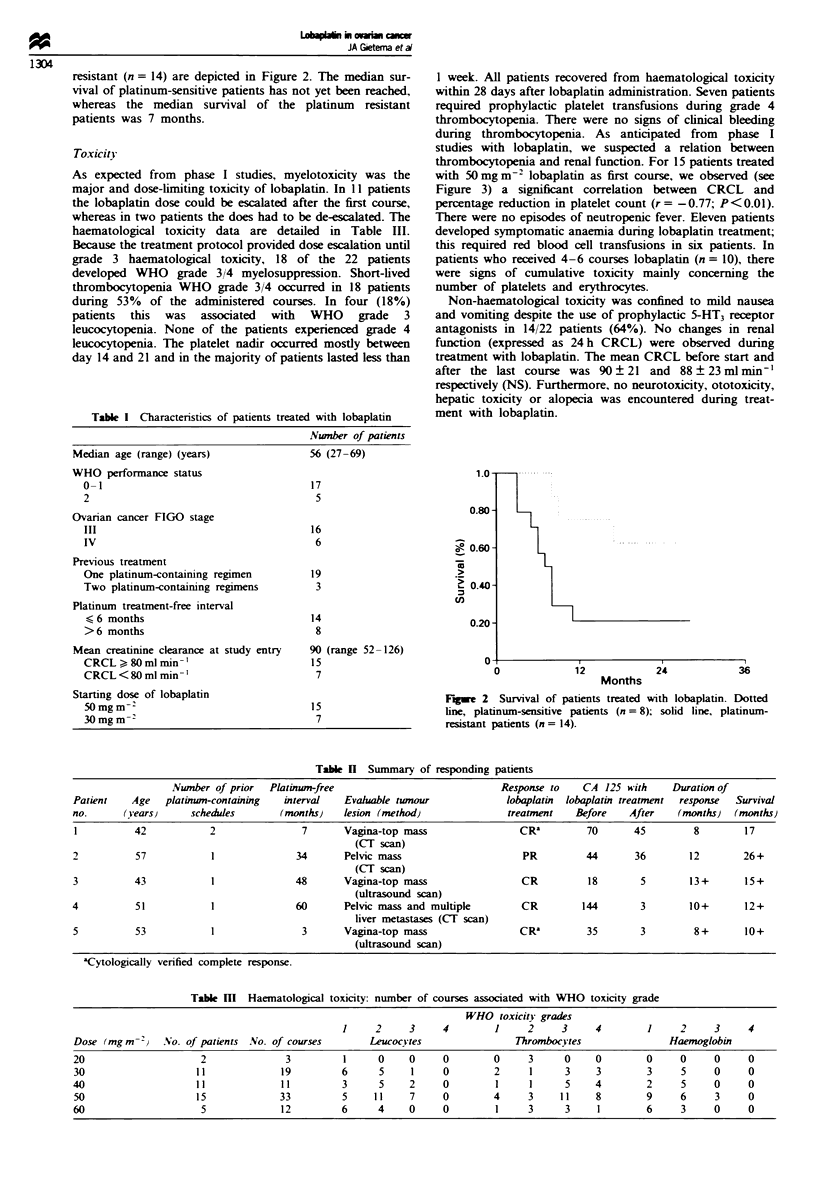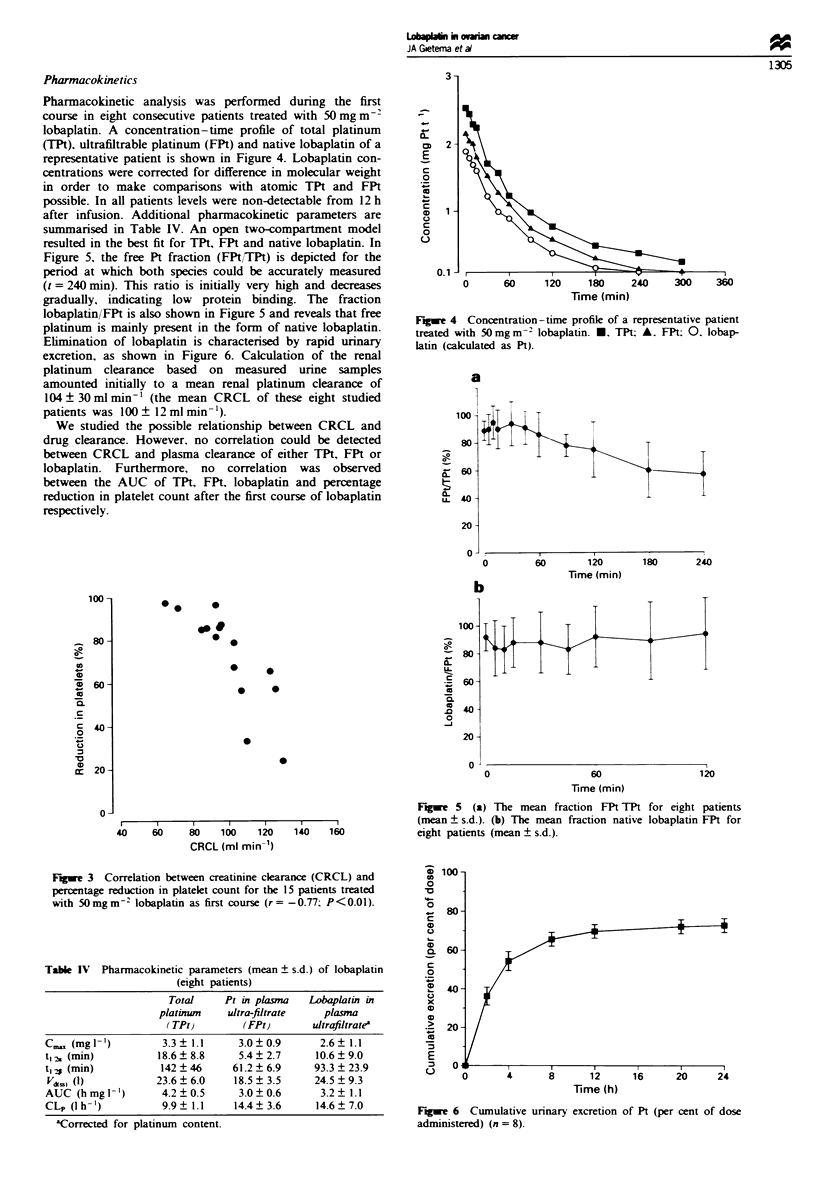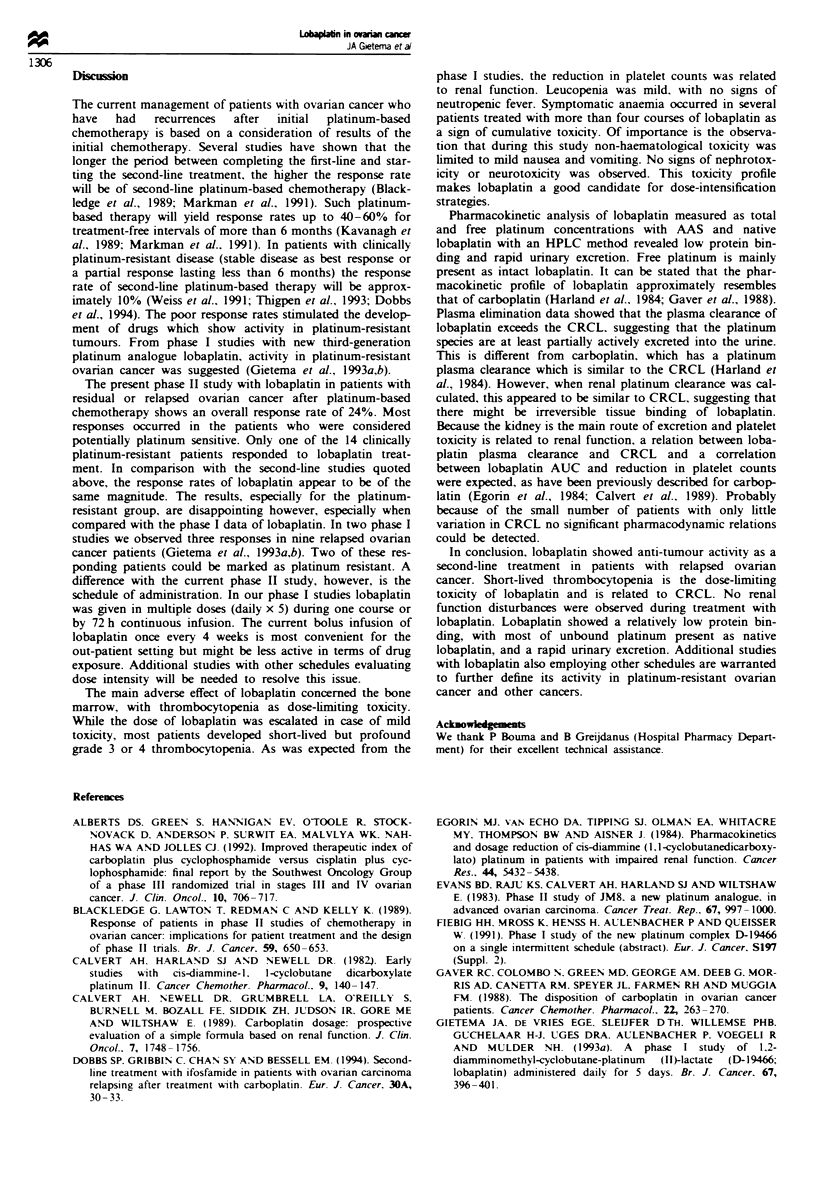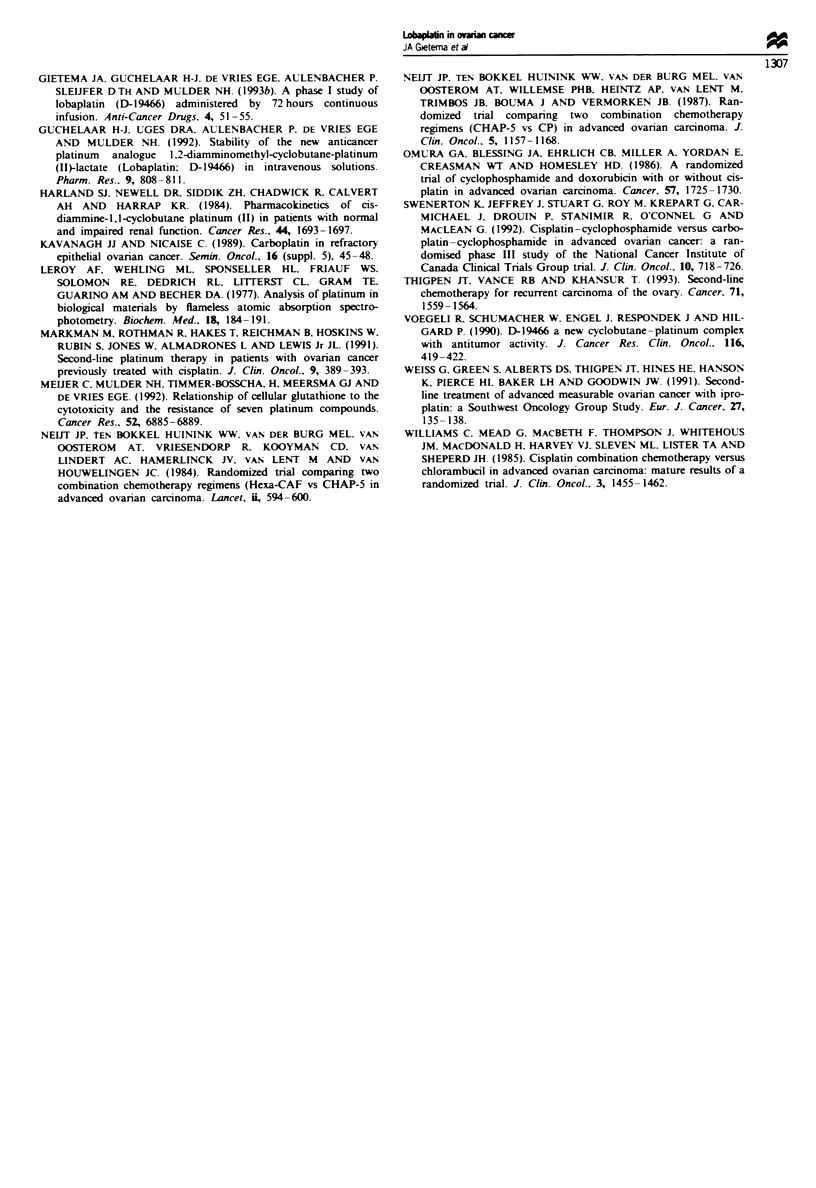# Phase II and pharmacokinetic study of lobaplatin in patients with relapsed ovarian cancer.

**DOI:** 10.1038/bjc.1995.252

**Published:** 1995-06

**Authors:** J. A. Gietema, G. J. Veldhuis, H. J. Guchelaar, P. H. Willemse, D. R. Uges, A. Cats, H. Boonstra, W. T. van der Graaf, D. T. Sleijfer, E. G. de Vries

**Affiliations:** Department of Medical Oncology, University Hospital Groningen, The Netherlands.

## Abstract

In phase I studies, lobaplatin showed activity in ovarian cancer patients pretreated with platinum. A phase II trial with lobaplatin was performed in patients with refractory or relapsed ovarian cancer to define activity and pharmacokinetics. Twenty-two patients were treated with lobaplatin administered as an intravenous bolus every 4 weeks. Dependent on creatinine clearance (CRCL) patients received 30 or 50 mg m-2 lobaplatin as the starting dose. Twenty-two patients received 78 courses (median 3, range 1-6). In eight patients total platinum (TPt) in plasma and urine, free platinum (FPt) in plasma ultrafiltrate (both measured by atomic absorption spectrometry) and lobaplatin in plasma ultrafiltrate measured (by high-performance liquid chromatography) were measured. Toxicity was confined to mild nausea and vomiting, mild leucocytopenia (WHO grade 3 in 18% of the courses), and renal function-related thrombocytopenia (WHO grade 3/4 in 53% of the courses). A correlation was found between CRCL and reduction in platelet count (r = -0.77; P < 0.01). No renal toxicity was encountered. Five of 21 evaluable patients (24%) achieved a response (four complete remissions and one partial remission). Remissions occurred mainly in patients who relapsed more than 6 months after primary treatment. The median survival from start of lobaplatin treatment was 8 months. The mean areas under the curve (AUCs) were 4.2 +/- 0.5, 3.0 +/- 0.6, and 3.2 +/- 1.1 h mgl-1 for TPt, FPt and lobaplatin respectively. The free platinum fraction (FPt/TPt) was initially very high, indicating low protein binding. FPt was essentially present as intact lobaplatin. Four hours after infusion 54 +/- 5% and 24 h after infusion 74 +/- 3% of the lobaplatin dose was excreted in the urine. In conclusion, lobaplatin is a platinum compound with anti-tumour activity in patients with relapsed ovarian cancer, especially in those who have platinum-sensitive tumours. The main toxicity of lobaplatin is thrombocytopenia and its dose should be corrected according to renal function.


British Journal of Cancer (1995) 71, 1302-1307

© 1995 Stockton Press All rights reserved 0007-0920/95 $12.00

### Phase II and pharmacokinetic study of lobaplatin in patients with relapsed ovarian cancer

JA Gietema[1], G-J Veldhuis[1], H-J Guchelaar[2], PHB Willemse[1], DRA Uges[2], A Cats[1], H Boonstra[3], WTA van der Graaf[1], D Th Sleijfer[1], EGE de Vries[1] and NH Mulder[1]

*Departments of [1]Medical Oncology, [2]Hospital Pharmacy and [3]Oncologic Gynaecology, University Hospital Groningen, The Netherlands.*

**Summary** In phase I studies, lobaplatin showed activity in ovarian cancer patients pretreated with platinum. A phase II trial with lobaplatin was performed in patients with refractory or relapsed ovarian cancer to define activity and pharmacokinetics. Twenty-two patients were treated with lobaplatin administered as an intravenous bolus every 4 weeks. Dependent on creatinine clearance (CRCL) patients received 30 or 50 mg $m^{-2}$ lobaplatin as the starting dose. Twenty-two patients received 78 courses (median 3, range 1-6). In eight patients total platinum (TPt) in plasma and urine, free platinum (FPt) in plasma ultrafiltrate (both measured by atomic absorption spectrometry) and lobaplatin in plasma ultrafiltrate measured (by high-performance liquid chromatography) were measured. Toxicity was confined to mild nausea and vomiting, mild leucocytopenia (WHO grade 3 in 18% of the courses), and renal function-related thrombocytopenia (WHO grade 3/4 in 53% of the courses). A correlation was found between CRCL and reduction in platelet count ($r = -0.77$; $P<0.01$). No renal toxicity was encountered. Five of 21 evaluable patients (24%) achieved a response (four complete remissions and one partial remission). Remissions occurred mainly in patients who relapsed more than 6 months after primary treatment. The median survival from start of lobaplatin treatment was 8 months. The mean areas under the curve (AUCs) were $4.2 \pm 0.5$, $3.0 \pm 0.6$, and $3.2 \pm 1.1$ h mg$l^{-1}$ for TPt, FPt and lobaplatin respectively. The free platinum fraction (FPt/TPt) was initially very high, indicating low protein binding. FPt was essentially present as intact lobaplatin. Four hours after infusion $54 \pm 5$% and 24 h after infusion $74 \pm 3$% of the lobaplatin dose was excreted in the urine. In conclusion, lobaplatin is a platinum compound with anti-tumour activity in patients with relapsed ovarian cancer, especially in those who have platinum-sensitive tumours. The main toxicity of lobaplatin is thrombocytopenia and its dose should be corrected according to renal function.

**Keywords:** ovarian cancer; phase II; lobaplatin

Over the last decade, treatment of patients with ovarian cancer has been dominated by cisplatin-containing regimens (Neijt *et al.*, 1984; Williams *et al.*, 1985; Omura *et al.*, 1986). Combinations of cisplatin with one single alkylating agent give equivalent results to three- or four-drug schedules, but appear to be less toxic (Neijt *et al.*, 1987). In recent years, the development of new drugs has been directed towards the development of platinum analogues that are equipotent but less toxic than the parent compound. Carboplatin has emerged as leading analogue in this respect with reduced nephrotoxicity, gastointestinal toxicity and neurotoxicity (Calvert *et al.*, 1982; Evans *et al.*, 1983). Myelotoxicity, especially thrombocytopenia, has been found to be the dose-limiting toxicity of carboplatin. Especially in ovarian cancer, carboplatin appears to have equivalent activity to cisplatin (Alberts *et al.*, 1992; Swenerton *et al.*, 1992).

An important direction in current research is to find new cisplatin analogues that are less toxic and more effective than second-generation analogues such as carboplatin. One of these compounds might be lobaplatin (1,2-diamminomethyl-cyclobutane-platinum (II)-lactate, D-19466) (Figure 1). Lobaplatin has a greater anti-tumour effect *in vitro* towards B16 melanoma and AH13s hepatoma than cisplatin (Voegeli *et al.*, 1990). This was also implied by experiments performed in two cell lines and their cisplatin-resistant sublines. In a small-cell lung carcinoma cell line ($GLC_4$) and its resistant subline ($GLC_4$-CDDP), lobaplatin showed full cross-resistance, whereas in another line, a human embryonal cancer cell line (Ntera2/D1), and its cisplatin-resistant subline (tera-CP), lobaplatin demonstrated no cross-resistance (Meijer *et al.*, 1992). *In vivo*, in mice bearing P388 leukaemia, administration of lobaplatin resulted in a greater increase in lifespan than cisplatin or carboplatin. In a cisplatin-resistant P388 tumour in which neither cisplatin nor carboplatin was able to inhibit the proliferation after transplantation, the survival of the animals was significantly prolonged by lobaplatin (Voegeli *et al.*, 1990). These preclinical *in vitro* and animal data suggest that the anti-tumour activity of lobaplatin is different from that of cisplatin and carboplatin and might be not cross-resistant.

In phase I studies with lobaplatin administered by different schedules (daily bolus infusion for 5 days, 72 h continues infusion and single bolus infusion) its main toxicity appeared to be on the bone marrow, and especially concerned thrombocytopenia (Fiebig *et al.*, 1991; Gietema *et al.*, 1993*a*,*b*). Gastrointestinal toxicity was mild and renal toxicity did not occur. In the daily bolus infusion for 5 days schedule the thrombocytopenia was clearly related with the renal function of the individual patients, resulting in different maximal tolerated dosages for different renal function cohorts (Gietema *et al.*, 1993*a*). In a one day bolus and a continuous infusion schedule the toxicity pattern was similar to that of a 5 day regimen. The optimal dosages of lobaplatin in the three outlined schedules for patients with a creatinine clearance

**Figure 1** Structure of lobaplatin.

Correspondence: NH Mulder, Department of Internal Medicine, Division of Medical Oncology, University Hospital Groningen, Oostersingel 59, 9713 EZ Groningen, The Netherlands
Received 30 September 1994; revised 23 December 1994; accepted 4 January 1995

(CRCL) above 80 ml $min^{-1}$ were between 45 and 70 mg $m^{-2}$. In phase I studies with lobaplatin tumour responses were seen in several patients with (partial) platinum-resistant ovarian carcinoma (Gietema *et al.*, 1993*a,b*).

The promising results from the phase I studies prompted us to undertake a phase II study using a single bolus infusion of lobaplatin in patients with residual or relapsed ovarian cancer after treatment with a platinum-based regimen. In addition, detailed analysis of the pharmacokinetics of lobaplatin was performed.

### Patients and methods

Twenty-two patients with FIGO stage III or IV ovarian cancer and measurable tumour lesions, recurrent or residual after prior platinum-based combination chemotherapy, were entered into this phase II study. To be eligible for this study, patients had to fulfil the following criteria: (a) age 18–75 years; (b) an estimated life expectancy of $\geqslant 3$ months; (c) a WHO performance status of $\leqslant 2$; (d) complete recovery from all toxic effects from prior treatments with a treatment-free interval of at least 4 weeks; (e) adequate bone marrow function (leucocyte count $\geqslant 3 \times 10^9\,l^{-1}$ and platelet count $\geqslant 100 \times 10^9\,l^{-1}$; (f) serum creatinine level $\leqslant 135\,\mu mol\,l^{-1}$ and a creatinine clearance (CRCL) $\geqslant 40$ ml $min^{-1}$; (g) serum bilirubin $\leqslant 30\,\mu mol\,l^{-1}$. This protocol was approved by the Medical Ethical Committee of the University Hospital Groningen, The Netherlands. Informed consent was obtained from all patients after they were informed of the investigational nature of this treatment.

Lobaplatin was supplied by ASTA Medica (Frankfurt, Germany). The dose of lobaplatin was dissolved in sterile water and reconstituted in 100 ml of 0.9% sodium chloride. Lobaplatin was administered as an i.v. bolus once every 4 weeks. The starting dose of lobaplatin depended on renal function expressed as creatinine clearance (CRCL). Patients with a CRCL $\geqslant 80$ ml $min^{-1}$ started with 50 mg $m^{-2}$ lobaplatin. Patients with a CRCL $<80$ ml $min^{-1}$ started with 30 mg $m^{-2}$. Dose escalation with 10 mg $m^{-2}$ was performed in case of haematological toxicity $\leqslant$ WHO grade 2. In the case of WHO grade 4 haematological toxicity the dose of the next cycle was de-escalated with 10 mg $m^{-2}$. For WHO grade 3 myelotoxicity no changes were made. This dose modification scheme would allow every patient to be treated on the maximum tolerated dose level of lobaplatin. Toxicity was evaluated according to WHO criteria. Prophylactic antiemetics, i.e. 5-$HT_3$ antagonists, were administered to all patients.

To be evaluable for response, patients had to receive at least two courses of lobaplatin. Tumour evaluations were performed at entry and after every two treatment cycles. A complete response (CR) was defined as a disappearance of all evidence of the tumour and no development of new lesions for at least 4 weeks. A partial response (PR) was defined as a decrease of at least 50% in the sum of the products of the largest perpendicular diameters of all measurable lesion. Stable disease meant a decrease within 50% or an increase of less than 25% in any measurable lesions. Progressive disease was defined as an increase of more than 25% of the lesions or the occurrence of any new lesions. Measurements of CA-125 were only used to support these response data. Patients were removed from the study in case of progressive disease and for severe non-haematological toxicity (WHO grade 3–4). A maximum of six cycles were given.

At entry and during each 28 days course, complete blood cell count, electrolytes, liver and renal function and glucose were measured on the treatment day and on days 14, 21, and 28. Twenty-four hour urinary creatinine clearances were performed twice before study entry, before each course and after the last course of lobaplatin.

Pharmacokinetic analysis was performed in eight consecutive patients during the first course of lobaplatin 50 mg $m^{-2}$. Blood samples were drawn on ice from the non-infused arm in heparinised glass tubes (Venoject, Omnilabo, Breda, The Netherlands) before infusion and at $t = 0$ (just after end of infusion) and 5, 10, 15, 30, 45, 60, 90, 120, 180, 240, 300, 720 and 1440 min after end of lobaplatin infusion. Samples were immediately centrifuged. Ultrafiltrate samples were prepared by centrifugation over an Amicon Centifree microparitition system provided with a YMT cut-off filter (MW $>$ 30 000 Da) (Product No. 4104, Amicon, Oosterhout, The Netherlands). The plasma and ultrafiltrate samples were stored at $-20$°C until analysis. Portions of urine were collected at six appropriate intervals during 24 h following the infusion and stored at $-20$°C until analysis. Platinum concentration in the samples was determined by flameless atomic absorption spectrometry (AAS) (Leroy *et al.*, 1977). The amount of platinum was determined using a model AA1275 atomic absorption spectrophotometer with a GTA95 graphite furnace and autosampler unit (Varian Techtron, Mulgrave, Victoria, Australia). Absorption was measured at 265.9 nm with a spectral bandwidth of 0.5 nm and deuterium background correction. This method has a limit of detection for platinum of 0.1 mg $l^{-1}$. Lobaplatin was determined by a high-performance liquid chromatographic (HPLC) method which has been described previously (Guchelaar *et al.*, 1992). This method has a limit of detection of lobaplatin of 0.2 mg $l^{-1}$. The plasma concentration–time data for the individual patients were subjected to analysis using a computer program for pharmacokinetic linear curve fitting (MWPharm, Mediware, Groningen, The Netherlands). The area under the plasma concentration–time curve (AUC) was calculated using the model-independent trapezoidal rule.

Regression analysis in this study was calculated by the method of least squares. Statistical analysis was performed with the Student's *t*-test. Only two-tailed $P$-values $<0.05$ were considered to be significant.

### Results

### *Patients*

The characteristics of patients entered in this phase II trial are outlined in Table I. The 22 patients received 78 courses (median 3, range 1–6). All patients were evaluable for toxicity and 21 also for response. Nineteen patients had been treated with one previous chemotherapy schedule, three patients had received two previous schedules.

Eight patients achieved a partial ($n = 3$) or complete response ($n = 5$) on previous therapy which lasted for more than 6 months. These patients were considered clinically platinum sensitive. The outcome of prior treatment of the remaining 14 patients was progressive disease ($n = 4$); stable disease ($n = 5$); or relapse within 6 months after a partial response ($n = 5$). The last patient group was considered to be clinically platinum resistant. The median platinum-free intervals of the platinum-sensitive and the platinum-resistant group were 30 months (range 7–60) and 2 months (range 2–6) respectively.

### *Response and survival*

Twenty-one patients were evaluable for response as one patient had progressive disease during the first course of lobaplatin. There were four complete responses and one partial response. The overall response rate was 24% (95% confidence interval 8–47%). Details of responders are summarised in Table II. Most responses occurred in pelvic masses; in two patients the response was cytologically verified. Remissions occurred more frequently in patients who were considered clinically platinum sensitive than in those who were platinum resistant: four (50%) *vs* one (8%) respectively. Median response duration was 10 months (range 8–13+ months). Two patients had stable disease and 14 patients had progressive disease during lobaplatin treatment.

The median survival from the date of starting chemotherapy with lobaplatin was 8 months (range 3–28). Survival of patients who were considered clinically platinum sensitive ($n = 8$) and of patients who were clinically platinum resistant ($n = 14$) are depicted in Figure 2. The median survival of platinum-sensitive patients has not yet been reached, whereas the median survival of the platinum resistant patients was 7 months.

### *Toxicity*

As expected from phase I studies, myelotoxicity was the major and dose-limiting toxicity of lobaplatin. In 11 patients the lobaplatin dose could be escalated after the first course, whereas in two patients the does had to be de-escalated. The haematological toxicity data are detailed in Table III. Because the treatment protocol provided dose escalation until grade 3 haematological toxicity, 18 of the 22 patients developed WHO grade 3/4 myelosuppression. Short-lived thrombocytopenia WHO grade 3/4 occurred in 18 patients during 53% of the administered courses. In four (18%) patients this was associated with WHO grade 3 leucocytopenia. None of the patients experienced grade 4 leucocytopenia. The platelet nadir occurred mostly between day 14 and 21 and in the majority of patients lasted less than 1 week. All patients recovered from haematological toxicity within 28 days after lobaplatin administration. Seven patients required prophylactic platelet transfusions during grade 4 thrombocytopenia. There were no signs of clinical bleeding during thrombocytopenia. As anticipated from phase I studies with lobaplatin, we suspected a relation between thrombocytopenia and renal function. For 15 patients treated with 50 mg $m^{-2}$ lobaplatin as first course, we observed (see Figure 3) a significant correlation between CRCL and percentage reduction in platelet count ($r = -0.77$; $P < 0.01$). There were no episodes of neutropenic fever. Eleven patients developed symptomatic anaemia during lobaplatin treatment; this required red blood cell transfusions in six patients. In patients who received 4–6 courses lobaplatin ($n = 10$), there were signs of cumulative toxicity mainly concerning the number of platelets and erythrocytes.

Non-haematological toxicity was confined to mild nausea and vomiting despite the use of prophylactic 5-$HT_3$ receptor antagonists in 14/22 patients (64%). No changes in renal function (expressed as 24 h CRCL) were observed during treatment with lobaplatin. The mean CRCL before start and after the last course was $90 \pm 21$ and $88 \pm 23$ ml $min^{-1}$ respectively (NS). Furthermore, no neurotoxicity, ototoxicity, hepatic toxicity or alopecia was encountered during treatment with lobaplatin.

**Table I** Characteristics of patients treated with lobaplatin

| | *Number of patients* |
|---|---|
| Median age (range) (years) | 56 (27–69) |
| WHO performance status | |
| 0–1 | 17 |
| 2 | 5 |
| Ovarian cancer FIGO stage | |
| III | 16 |
| IV | 6 |
| Previous treatment | |
| One platinum-containing regimen | 19 |
| Two platinum-containing regimens | 3 |
| Platinum treatment-free interval | |
| $\leqslant 6$ months | 14 |
| $> 6$ months | 8 |
| Mean creatinine clearance at study entry | 90 (range 52–126) |
| CRCL $\geqslant 80$ ml $min^{-1}$ | 15 |
| CRCL $< 80$ ml $min^{-1}$ | 7 |
| Starting dose of lobaplatin | |
| 50 mg $m^{-2}$ | 15 |
| 30 mg $m^{-2}$ | 7 |

**Figure 2** Survival of patients treated with lobaplatin. Dotted line, platinum-sensitive patients ($n = 8$); solid line, platinum-resistant patients ($n = 14$).

**Table II** Summary of responding patients

| Patient no. | Age (years) | Number of prior platinum-containing schedules | Platinum-free interval (months) | Evaluable tumour lesion (method) | Response to lobaplatin treatment | CA 125 with lobaplatin treatment Before | After | Duration of response (months) | Survival (months) |
|---|---|---|---|---|---|---|---|---|---|
| 1 | 42 | 2 | 7 | Vagina-top mass (CT scan) | CR[a] | 70 | 45 | 8 | 17 |
| 2 | 57 | 1 | 34 | Pelvic mass (CT scan) | PR | 44 | 36 | 12 | 26+ |
| 3 | 43 | 1 | 48 | Vagina-top mass (ultrasound scan) | CR | 18 | 5 | 13+ | 15+ |
| 4 | 51 | 1 | 60 | Pelvic mass and multiple liver metastases (CT scan) | CR | 144 | 3 | 10+ | 12+ |
| 5 | 53 | 1 | 3 | Vagina-top mass (ultrasound scan) | CR[a] | 35 | 3 | 8+ | 10+ |

[a]Cytologically verified complete response.

**Table III** Haematological toxicity: number of courses associated with WHO toxicity grade

| | | | *WHO toxicity grades* | | | | | | | | | | | |
|---|---|---|---|---|---|---|---|---|---|---|---|---|---|---|
| | | | *Leucocytes* | | | | *Thrombocytes* | | | | *Haemoglobin* | | | |
| *Dose (mg $m^{-2}$)* | *No. of patients* | *No. of courses* | *1* | *2* | *3* | *4* | *1* | *2* | *3* | *4* | *1* | *2* | *3* | *4* |
| 20 | 2 | 3 | 1 | 0 | 0 | 0 | 0 | 3 | 0 | 0 | 0 | 0 | 0 | 0 |
| 30 | 11 | 19 | 6 | 5 | 1 | 0 | 2 | 1 | 3 | 3 | 3 | 5 | 0 | 0 |
| 40 | 11 | 11 | 3 | 5 | 2 | 0 | 1 | 1 | 5 | 4 | 2 | 5 | 0 | 0 |
| 50 | 15 | 33 | 5 | 11 | 7 | 0 | 4 | 3 | 11 | 8 | 9 | 6 | 3 | 0 |
| 60 | 5 | 12 | 6 | 4 | 0 | 0 | 1 | 3 | 3 | 1 | 6 | 3 | 0 | 0 |

### Pharmacokinetics

Pharmacokinetic analysis was performed during the first course in eight consecutive patients treated with 50 mg m$^{-2}$ lobaplatin. A concentration-time profile of total platinum (TPt), ultrafiltrable platinum (FPt) and native lobaplatin of a representative patient is shown in Figure 4. Lobaplatin concentrations were corrected for difference in molecular weight in order to make comparisons with atomic TPt and FPt possible. In all patients levels were non-detectable from 12 h after infusion. Additional pharmacokinetic parameters are summarised in Table IV. An open two-compartment model resulted in the best fit for TPt, FPt and native lobaplatin. In Figure 5, the free Pt fraction (FPt/TPt) is depicted for the period at which both species could be accurately measured ($t$ = 240 min). This ratio is initially very high and decreases gradually, indicating low protein binding. The fraction lobaplatin/FPt is also shown in Figure 5 and reveals that free platinum is mainly present in the form of native lobaplatin. Elimination of lobaplatin is characterised by rapid urinary excretion, as shown in Figure 6. Calculation of the renal platinum clearance based on measured urine samples amounted initially to a mean renal platinum clearance of 104 ± 30 ml min$^{-1}$ (the mean CRCL of these eight studied patients was 100 ± 12 ml min$^{-1}$).

We studied the possible relationship between CRCL and drug clearance. However, no correlation could be detected between CRCL and plasma clearance of either TPt, FPt or lobaplatin. Furthermore, no correlation was observed between the AUC of TPt, FPt, lobaplatin and percentage reduction in platelet count after the first course of lobaplatin respectively.

**Figure 3** Correlation between creatinine clearance (CRCL) and percentage reduction in platelet count for the 15 patients treated with 50 mg m$^{-2}$ lobaplatin as first course ($r = -0.77$; $P<0.01$).

**Table IV** Pharmacokinetic parameters (mean ± s.d.) of lobaplatin (eight patients)

| | *Total platinum (TPt)* | *Pt in plasma ultra-filtrate (FPt)* | *Lobaplatin in plasma ultrafiltrate*[a] |
|---|---|---|---|
| $C_{max}$ (mg l$^{-1}$) | 3.3 ± 1.1 | 3.0 ± 0.9 | 2.6 ± 1.1 |
| $t_{1/2\alpha}$ (min) | 18.6 ± 8.8 | 5.4 ± 2.7 | 10.6 ± 9.0 |
| $t_{1/2\beta}$ (min) | 142 ± 46 | 61.2 ± 6.9 | 93.3 ± 23.9 |
| $V_{d(ss)}$ (l) | 23.6 ± 6.0 | 18.5 ± 3.5 | 24.5 ± 9.3 |
| AUC (h mg l$^{-1}$) | 4.2 ± 0.5 | 3.0 ± 0.6 | 3.2 ± 1.1 |
| $CL_P$ (l h$^{-1}$) | 9.9 ± 1.1 | 14.4 ± 3.6 | 14.6 ± 7.0 |

[a]Corrected for platinum content.

**Figure 4** Concentration-time profile of a representative patient treated with 50 mg m$^{-2}$ lobaplatin. ■, TPt; ▲, FPt; ○, lobaplatin (calculated as Pt).

**Figure 5** (**a**) The mean fraction FPt/TPt for eight patients (mean ± s.d.). (**b**) The mean fraction native lobaplatin/FPt for eight patients (mean ± s.d.).

**Figure 6** Cumulative urinary excretion of Pt (per cent of dose administered) ($n$ = 8).

### Discussion

The current management of patients with ovarian cancer who have had recurrences after initial platinum-based chemotherapy is based on a consideration of results of the initial chemotherapy. Several studies have shown that the longer the period between completing the first-line and starting the second-line treatment, the higher the response rate will be of second-line platinum-based chemotherapy (Blackledge *et al*., 1989; Markman *et al*., 1991). Such platinum-based therapy will yield response rates up to 40-60% for treatment-free intervals of more than 6 months (Kavanagh *et al*., 1989; Markman *et al*., 1991). In patients with clinically platinum-resistant disease (stable disease as best response or a partial response lasting less than 6 months) the response rate of second-line platinum-based therapy will be approximately 10% (Weiss *et al*., 1991; Thigpen *et al*., 1993; Dobbs *et al*., 1994). The poor response rates stimulated the development of drugs which show activity in platinum-resistant tumours. From phase I studies with new third-generation platinum analogue lobaplatin, activity in platinum-resistant ovarian cancer was suggested (Gietema *et al*., 1993*a*,*b*).

The present phase II study with lobaplatin in patients with residual or relapsed ovarian cancer after platinum-based chemotherapy shows an overall response rate of 24%. Most responses occurred in the patients who were considered potentially platinum sensitive. Only one of the 14 clinically platinum-resistant patients responded to lobaplatin treatment. In comparison with the second-line studies quoted above, the response rates of lobaplatin appear to be of the same magnitude. The results, especially for the platinum-resistant group, are disappointing however, especially when compared with the phase I data of lobaplatin. In two phase I studies we observed three responses in nine relapsed ovarian cancer patients (Gietema *et al*., 1993*a*,*b*). Two of these responding patients could be marked as platinum resistant. A difference with the current phase II study, however, is the schedule of administration. In our phase I studies lobaplatin was given in multiple doses (daily × 5) during one course or by 72 h continuous infusion. The current bolus infusion of lobaplatin once every 4 weeks is most convenient for the out-patient setting but might be less active in terms of drug exposure. Additional studies with other schedules evaluating dose intensity will be needed to resolve this issue.

The main adverse effect of lobaplatin concerned the bone marrow, with thrombocytopenia as dose-limiting toxicity. While the dose of lobaplatin was escalated in case of mild toxicity, most patients developed short-lived but profound grade 3 or 4 thrombocytopenia. As was expected from the phase I studies, the reduction in platelet counts was related to renal function. Leucopenia was mild, with no signs of neutropenic fever. Symptomatic anaemia occurred in several patients treated with more than four courses of lobaplatin as a sign of cumulative toxicity. Of importance is the observation that during this study non-haematological toxicity was limited to mild nausea and vomiting. No signs of nephrotoxicity or neurotoxicity was observed. This toxicity profile makes lobaplatin a good candidate for dose-intensification strategies.

Pharmacokinetic analysis of lobaplatin measured as total and free platinum concentrations with AAS and native lobaplatin with an HPLC method revealed low protein binding and rapid urinary excretion. Free platinum is mainly present as intact lobaplatin. It can be stated that the pharmacokinetic profile of lobaplatin approximately resembles that of carboplatin (Harland *et al*., 1984; Gaver *et al*., 1988). Plasma elimination data showed that the plasma clearance of lobaplatin exceeds the CRCL, suggesting that the platinum species are at least partially actively excreted into the urine. This is different from carboplatin, which has a platinum plasma clearance which is similar to the CRCL (Harland *et al*., 1984). However, when renal platinum clearance was calculated, this appeared to be similar to CRCL, suggesting that there might be irreversible tissue binding of lobaplatin. Because the kidney is the main route of excretion and platelet toxicity is related to renal function, a relation between lobaplatin plasma clearance and CRCL and a correlation between lobaplatin AUC and reduction in platelet counts were expected, as have been previously described for carboplatin (Egorin *et al*., 1984; Calvert *et al*., 1989). Probably because of the small number of patients with only little variation in CRCL no significant pharmacodynamic relations could be detected.

In conclusion, lobaplatin showed anti-tumour activity as a second-line treatment in patients with relapsed ovarian cancer. Short-lived thrombocytopenia is the dose-limiting toxicity of lobaplatin and is related to CRCL. No renal function disturbances were observed during treatment with lobaplatin. Lobaplatin showed a relatively low protein binding, with most of unbound platinum present as native lobaplatin, and a rapid urinary excretion. Additional studies with lobaplatin also employing other schedules are warranted to further define its activity in platinum-resistant ovarian cancer and other cancers.

**Acknowledgements**
We thank P Bouma and B Greijdanus (Hospital Pharmacy Department) for their excellent technical assistance.

### References

ALBERTS DS, GREEN S, HANNIGAN EV, O'TOOLE R, STOCK-NOVACK D, ANDERSON P, SURWIT EA, MALVLYA WK, NAHHAS WA AND JOLLES CJ. (1992). Improved therapeutic index of carboplatin plus cyclophosphamide versus cisplatin plus cyclophosphamide: final report by the Southwest Oncology Group of a phase III randomized trial in stages III and IV ovarian cancer. *J. Clin. Oncol.*, **10**, 706-717.

BLACKLEDGE G, LAWTON T, REDMAN C AND KELLY K. (1989). Response of patients in phase II studies of chemotherapy in ovarian cancer: implications for patient treatment and the design of phase II trials. *Br. J. Cancer*, **59**, 650-653.

CALVERT AH, HARLAND SJ AND NEWELL DR. (1982). Early studies with cis-diammine-1, 1-cyclobutane dicarboxylate platinum II. *Cancer Chemother. Pharmacol.*, **9**, 140-147.

CALVERT AH, NEWELL DR, GRUMBRELL LA, O'REILLY S, BURNELL M, BOZALL FE, SIDDIK ZH, JUDSON IR, GORE ME AND WILTSHAW E. (1989). Carboplatin dosage: prospective evaluation of a simple formula based on renal function. *J. Clin. Oncol.*, **7**, 1748-1756.

DOBBS SP, GRIBBIN C, CHAN SY AND BESSELL EM. (1994). Second-line treatment with ifosfamide in patients with ovarian carcinoma relapsing after treatment with carboplatin. *Eur. J. Cancer*, **30A**, 30-33.

EGORIN MJ, VAN ECHO DA, TIPPING SJ, OLMAN EA, WHITACRE MY, THOMPSON BW AND AISNER J. (1984). Pharmacokinetics and dosage reduction of cis-diammine (1,1-cyclobutanedicarboxylato) platinum in patients with impaired renal function. *Cancer Res.*, **44**, 5432-5438.

EVANS BD, RAJU KS, CALVERT AH, HARLAND SJ AND WILTSHAW E. (1983). Phase II study of JM8, a new platinum analogue, in advanced ovarian carcinoma. *Cancer Treat. Rep.*, **67**, 997-1000.

FIEBIG HH, MROSS K, HENSS H, AULENBACHER P AND QUEISSER W. (1991). Phase I study of the new platinum complex D-19466 on a single intermittent schedule (abstract). *Eur. J. Cancer*, **S197** (Suppl. 2).

GAVER RC, COLOMBO N, GREEN MD, GEORGE AM, DEEB G, MORRIS AD, CANETTA RM, SPEYER JL, FARMEN RH AND MUGGIA FM. (1988). The disposition of carboplatin in ovarian cancer patients. *Cancer Chemother. Pharmacol.*, **22**, 263-270.

GIETEMA JA, DE VRIES EGE, SLEIJFER DTH, WILLEMSE PHB, GUCHELAAR H-J, UGES DRA, AULENBACHER P, VOEGELI R AND MULDER NH. (1993*a*). A phase I study of 1,2-diamminomethyl-cyclobutane-platinum (II)-lactate (D-19466; lobaplatin) administered daily for 5 days. *Br. J. Cancer*, **67**, 396-401.

GIETEMA JA, GUCHELAAR H-J, DE VRIES EGE, AULENBACHER P, SLEIJFER D TH AND MULDER NH. (1993*b*). A phase I study of lobaplatin (D-19466) administered by 72 hours continuous infusion. *Anti-Cancer Drugs*, **4**, 51–55.

GUCHELAAR H-J, UGES DRA, AULENBACHER P, DE VRIES EGE AND MULDER NH. (1992). Stability of the new anticancer platinum analogue 1,2-diamminomethyl-cyclobutane-platinum (II)-lactate (Lobaplatin; D-19466) in intravenous solutions. *Pharm. Res.*, **9**, 808–811.

HARLAND SJ, NEWELL DR, SIDDIK ZH, CHADWICK R, CALVERT AH AND HARRAP KR. (1984). Pharmacokinetics of cis-diammine-1,1-cyclobutane platinum (II) in patients with normal and impaired renal function. *Cancer Res.*, **44**, 1693–1697.

KAVANAGH JJ AND NICAISE C. (1989). Carboplatin in refractory epithelial ovarian cancer. *Semin. Oncol.*, **16** (suppl. 5), 45–48.

LEROY AF, WEHLING ML, SPONSELLER HL, FRIAUF WS, SOLOMON RE, DEDRICH RL, LITTERST CL, GRAM TE, GUARINO AM AND BECHER DA. (1977). Analysis of platinum in biological materials by flameless atomic absorption spectrophotometry. *Biochem. Med.*, **18**, 184–191.

MARKMAN M, ROTHMAN R, HAKES T, REICHMAN B, HOSKINS W, RUBIN S, JONES W, ALMADRONES L AND LEWIS Jr JL. (1991). Second-line platinum therapy in patients with ovarian cancer previously treated with cisplatin. *J. Clin. Oncol.*, **9**, 389–393.

MEIJER C, MULDER NH, TIMMER-BOSSCHA, H, MEERSMA GJ AND DE VRIES EGE. (1992). Relationship of cellular glutathione to the cytotoxicity and the resistance of seven platinum compounds. *Cancer Res.*, **52**, 6885–6889.

NEIJT JP, TEN BOKKEL HUININK WW, VAN DER BURG MEL, VAN OOSTEROM AT, VRIESENDORP R, KOOYMAN CD, VAN LINDERT AC, HAMERLINCK JV, VAN LENT M AND VAN HOUWELINGEN JC. (1984). Randomized trial comparing two combination chemotherapy regimens (Hexa-CAF vs CHAP-5 in advanced ovarian carcinoma. *Lancet*, **ii**, 594–600.

NEIJT JP, TEN BOKKEL HUININK WW, VAN DER BURG MEL, VAN OOSTEROM AT, WILLEMSE PHB, HEINTZ AP, VAN LENT M, TRIMBOS JB, BOUMA J AND VERMORKEN JB. (1987). Randomized trial comparing two combination chemotherapy regimens (CHAP-5 vs CP) in advanced ovarian carcinoma. *J. Clin. Oncol.*, **5**, 1157–1168.

OMURA GA, BLESSING JA, EHRLICH CB, MILLER A, YORDAN E, CREASMAN WT AND HOMESLEY HD. (1986). A randomized trial of cyclophosphamide and doxorubicin with or without cisplatin in advanced ovarian carcinoma. *Cancer*, **57**, 1725–1730.

SWENERTON K, JEFFREY J, STUART G, ROY M, KREPART G, CARMICHAEL J, DROUIN P, STANIMIR R, O'CONNEL G AND MACLEAN G. (1992). Cisplatin–cyclophosphamide versus carboplatin–cyclophosphamide in advanced ovarian cancer: a randomised phase III study of the National Cancer Institute of Canada Clinical Trials Group trial. *J. Clin. Oncol.*, **10**, 718–726.

THIGPEN JT, VANCE RB AND KHANSUR T. (1993). Second-line chemotherapy for recurrent carcinoma of the ovary. *Cancer*, **71**, 1559–1564.

VOEGELI R, SCHUMACHER W, ENGEL J, RESPONDEK J AND HILGARD P. (1990). D-19466 a new cyclobutane–platinum complex with antitumor activity. *J. Cancer Res. Clin. Oncol.*, **116**, 419–422.

WEISS G, GREEN S, ALBERTS DS, THIGPEN JT, HINES HE, HANSON K, PIERCE HI, BAKER LH AND GOODWIN JW. (1991). Second-line treatment of advanced measurable ovarian cancer with iproplatin: a Southwest Oncology Group Study. *Eur. J. Cancer*, **27**, 135–138.

WILLIAMS C, MEAD G, MACBETH F, THOMPSON J, WHITEHOUS JM, MACDONALD H, HARVEY VJ, SLEVEN ML, LISTER TA AND SHEPERD JH. (1985). Cisplatin combination chemotherapy versus chlorambucil in advanced ovarian carcinoma: mature results of a randomized trial. *J. Clin. Oncol.*, **3**, 1455–1462.